# Scale-up of biomass production by *Methanococcus maripaludis*

**DOI:** 10.3389/fmicb.2022.1031131

**Published:** 2022-11-23

**Authors:** Hayk Palabikyan, Aquilla Ruddyard, Lara Pomper, David Novak, Barbara Reischl, Simon K.-M. R. Rittmann

**Affiliations:** ^1^Archaea Physiology & Biotechnology Group, Department of Functional and Evolutionary Ecology, Universität Wien, Vienna, Austria; ^2^Arkeon GmbH, Tulln a.d. Donau, Austria; ^3^Department of Biochemistry, Faculty of Science, Masaryk University, Brno, Czechia

**Keywords:** archaea biotechnology, bioreactor, bioprocess, fed-batch, anaerobe, methanogen

## Abstract

The development of a sustainable energy economy is one of the great challenges in the current times of climate crisis and growing energy demands. Industrial production of the fifth-generation biofuel methane by microorganisms has the potential to become a crucial biotechnological milestone of the post fossil fuel era. Therefore, reproducible cultivation and scale-up of methanogenic archaea (methanogens) is essential for enabling biomass generation for fundamental studies and for defining peak performance conditions for bioprocess development. This study provides a comprehensive revision of established and optimization of novel methods for the cultivation of the model organism *Methanococcus maripaludis* S0001. In closed batch mode, 0.05 L serum bottles cultures were gradually replaced by 0.4 L Schott bottle cultures for regular biomass generation, and the time for reaching peak optical density (OD_578_) values was reduced in half. In 1.5 L reactor cultures, various agitation, harvesting and transfer methods were compared resulting in a specific growth rate of 0.16 h^−1^ and the highest recorded OD_578_ of 3.4. Finally, a 300-fold scale-up from serum bottles was achieved by growing *M*. *maripaludis* for the first time in a 22 L stainless steel bioreactor with 15 L working volume. Altogether, the experimental approaches described in this study contribute to establishing methanogens as essential organisms in large-scale biotechnology applications, a crucial stage of an urgently needed industrial evolution toward sustainable biosynthesis of energy and high value products.

## Introduction

The global impact of a constantly expanding human civilization could be addressed by a proportionally accelerated transformation of the technological progress. Biotechnological advances utilizing natural and recombinant microorganisms, like methanogenic archaea (methanogens), can provide various solutions for decentralized sustainable energy manufacturing, such as biomethanation processes, which utilize methanogens with industrially relevant growth characteristics and high volumetric methane (CH_4_) biosynthesis (Seifert et al., 2014; Azim et al., 2017; Mauerhofer et al., 2018; Rittmann et al., 2018). Biological CH_4_ production from carbon dioxide (CO_2_) (CO_2_-BMP) can be applied to establish CH_4_ as a CO_2_-neutral biofuel (Porqueras et al., 2012) of the post-fossil fuel era, as CH_4_ can be incorporated into existing storage and transportation infrastructures for natural gas (Mauerhofer et al., 2018). In fact, methanogens have already been utilized in large scale anaerobic digestion for biogas production (Guebitz et al., 2015) and for conversion and storage of energy from renewable sources into CH_4_ (Götz et al., 2016).

Methanogens are a remarkable group of organisms within the domain Archaea (Cavicchioli, 2011; Borrel et al., 2013). Methanogenesis might have emerged billions of years ago under primordial conditions in hydrothermal vents as one of the most ancient metabolisms (Ueno et al., 2006; Martin et al., 2008). Today, methanogens have been found in almost every anoxic environment on the planet and are responsible for the final stage of biomass mineralization by producing CH_4_ under anaerobic conditions (Liu and Whitman, 2008; Thauer et al., 2008; Jabłoński et al., 2015). By biosynthesizing roughly 1 Gt of the potent greenhouse gas CH_4_ annually (Howarth et al., 2011; IPCC, 2013), methanogens have become a crucial subject of studies, due to their essential role in the global carbon cycle and their ecological significance.

*Methanococcus maripaludis* is an autotrophic, hydrogenotrophic, methanogenic mesophile which grows in mineral medium at 37°C (Jones et al., 1983; Keswani et al., 1996). *M*. *maripaludis* requires solely CO_2_ as a carbon source (Zellner and Winter, 1987) but it can additionally utilize acetate for biomass synthesis (Shieh and Whitman, 1987; Azim et al., 2018). For energy production, molecular hydrogen (H_2_) or formate are used as an electron source (Shieh and Whitman, 1988; Costa et al., 2013; Rother and Whitman, 2019). A thin S-layer has been described for *M*. *maripaludis* (Jarrell and Koval, 1989; Jarrell et al., 2010), which implies that *M*. *maripaludis* is presumably a rather fragile organism when it is exposed to low osmolarity buffers (Jones et al., 1985) or to detergents (Jones et al., 1983). Nevertheless, the cellular structure of *M*. *maripaludis* allows straightforward manipulation by classical molecular biology techniques.

Today, *M*. *maripaludis* is one of the mostly studied model organisms among obligate hydrogenotrophic methanogens (Goyal et al., 2016; Richards et al., 2016). It can be transformed (Tumbula et al., 1994) with various shuttle vectors (Argyle et al., 1996; Sarmiento et al., 2011; Walters et al., 2011), and its genome can be edited by integrative plasmids (Stathopoulos et al., 2001; Lie and Leigh, 2007) or by a markerless mutagenesis procedure (Moore and Leigh, 2005). Furthermore, two distinct CRISPR-mediated genome editing systems have been successfully established in *M*. *maripaludis* (Bao et al., 2022; Li et al., 2022). The extensive molecular toolbox has been used for diverse studies of the physiology of methanogens, such as the molecular architecture of the methyl coenzyme M reductase (Lyu et al., 2018). Furthermore, *M*. *maripaludis* S0001 has been metabolically engineered as a cell factory for the production of high value products, such as geraniol (Lyu et al., 2016) and the bioplastic polymer polyhydroxybutyrate (Thevasundaram et al., 2022). Nevertheless, there is much space for methodological advances which would allow *M*. *maripaludis* to be further established as model organism with a recognizable impact on the global biotechnology sector.

A reproducible pipeline for scaling up the cultivation of *M*. *maripaludis* has been a missing link that has restricted this promising species to a laboratory-scale subject of studies. Even though various techniques have been used for the cultivation of *M*. *maripaludis* in closed batch mode (Balch et al., 1979; Sarmiento et al., 2011; Goyal et al., 2015), they are all limiting culture volumes in milliliter ranges and alter physiological footprints due to a discontinuous substrate supplementation. Nevertheless, a flexible and cost-efficient formate-based 1.5 L system in closed batch mode has been developed for formate utilizing methanogens, which allows sufficient biomass generation for analytical studies and eliminates common challenges of H_2_/CO_2_-supplied methods (Long et al., 2017).

In contrast, a fed-batch mode of cultivation allows continuous addition of feed for growth, larger culture volumes and more sophisticated control of conditions. Nevertheless, only handful of notable studies have applied chemostat-like systems (Haydock et al., 2004; Hendrickson et al., 2007, 2008; Costa et al., 2013; Müller et al., 2021) or carried out scale-up of pure cultures (Walters and Chong, 2017) for biomass generation by of *M*. *maripaludis*. All of them have been focused on other fundamental questions and have not developed a detailed pipeline for continuous transfer and scale-up of *M*. *maripaludis* cultures. Even though larger cultivation volumes of 10 L have been developed decades ago (Shieh and Whitman, 1988), no follow up studies have optimized peak performance conditions for the cultivation of *M*. *maripaludis* in industrially relevant scales.

Building up from established techniques for the anaerobic cultivation of methanogens (Azim et al., 2017, 2018; Taubner and Rittmann, 2016) this study initiates the completion of a missing chapter in the study of *M*. *maripaludis* by designing an experimental approach for rapid biomass production by various cultivation methods.

## Materials and methods

### Microorganisms and media

*Methanococcus maripaludis* S0001 was used in all experiments. The organism was provided by William Barny Whitman, University of Georgia, United States. For all experiments and cultivation modes a reduced liquid 141 medium (DSMZ 141a) with the following composition was used: (L^−1^): 0.14 g CaCl_2_·2H_2_O, 0.34 g KCl, 4 g MgCl_2_·6H_2_O, 0.25 g NH_4_Cl, 18.09 NaCl, 0.14 g K_2_HPO_4_, 3.45 g MgSO_4_·7H_2_O, 10 mL Modified Wolin’s mineral solution (100×), 2 mL (0.1% w/v) Fe(NH_4_)_2_(SO_4_)_2_·6H_2_O (0.1% w/v). The 100× Modified Wolin’s mineral solution was prepared by dissolving 1.5 g Nitrilotriacetic acid in ddH_2_O and adjusting pH to 6.5 with KOH. Then the following reagents were added (L^−1^): 3 g MgSO_4_·7H_2_O, 0.585 g MnCl_2_·4H_2_O, 1 g NaCl, 0.18 g CoSO_4_·7H_2_O, 0.1 g FeSO_4_·7H_2_O, 0.1 g CaCl_2_·2H_2_O, 0.18 g ZnSO_4_·7H_2_O, 0.02 g KAI(SO_4_)_2_·12H_2_O, 0.006 g CuSO_4_, 0.01 g H_3_BO_3_, 0.01 g Na_2_MoO_4_·2H_2_O, 0.0003 g Na_2_SeO_3_·5H_2_O, 0.03 g NiCl_2_·6H_2_O, 0.0004 g NaWO_4_·2H_2_O. Finally, the pH was adjusted to 7.0 with KOH. The Fe(NH_4_)_2_(SO_4_)_2_·6H_2_O solution had the following composition (L^−1^): 0.00709 g FeSO_4_·7H_2_O and 0.00337 g (NH_4_)_2_SO_4_. After of the medium was made anaerobic and sterilized, it was supplemented with 20 mL L^−1^ of sodium acetate (0.61 mol L^−1^) and 4 mL L^−1^ of Na_2_S·9H_2_O (0.5 mol L^−1^) prior to inoculation. The following gasses were purchased from Air Liquide (Air Liquide GmbH, Schwechat, Austria) and used for cultivations: H_2_/CO_2_ (4:1 mix), H_2_ (≥99.999 Vol.-%) and CO_2_ (≥99.995 Vol.-%). Due to the combustible properties of those gasses, all experiments within this study were caried out in designated anaerobic facilities, equipped with sensors and gas alarm systems where the use of open fire is forbidden.

### Closed batch

#### 0.05 L serum bottle cultures

Serum bottles (SB) have total and culture volumes of 120 mL and 50 mL, respectively, and are hereafter referred as 0.05 L SB cultures. They were filled with 141 medium (48 mL) and sealed with butyl rubber stoppers (20 mm; CLS-3409-14; Chemglass Life Sciences, Vineland, NJ, United States) and aluminium crimp caps (20 mm; Ochs Laborbedarf, Bovenden, Germany). Bottles were made anaerobic by drawing vacuum (4×) and pressurizing (5× 1.0 bar) with H_2_/CO_2_ gas mixture (4:1). SB were sterilized by autoclaving, and each replicate was complemented with 1 mL sodium acetate (0.61 mol L^−1^) and 0.2 mL Na_2_S·9H_2_O (0.5 mol L^−1^) to a final volume of 49.2 mL prior to inoculation. Inoculation was carried out with 0.8 mL of pre-culture (1.6% (v/v)) in an anaerobic glove box (Coy Laboratory Products, Grass Lake, United States).

#### 0.4 L Schott bottle cultures

Schott bottles (SCB, DURAN® pressure plus GL-45; DWK Life Sciences, Mainz, Germany) have total and working volumes of 1,000 mL and 416.67 mL, respectively, and are hereafter referred as 0.4 L SCB cultures. They were filled with 141 medium (400 mL) and sealed with butyl rubber stoppers (40 mm; 444704, Glasgerätebau Ochs, Bovenden, Germany) and PBT screw caps (54 mm; DWK Life Sciences, Mainz, Germany). After they were made anaerobic and sterilized, each replicate was complemented with 8.33 mL sodium acetate (0.61 mol L^−1^) and 1.67 mL Na_2_S·9H_2_O (0.5 mol L^−1^) to a final volume of 410 mL prior to inoculation. Inoculation was carried out with 6.67 mL of pre-culture [1.6% (v/v)] in an anaerobic glove box (Coy Laboratory Products, Grass Lake, United States).

#### Cultivation and analysis

For inoculation, sampling and gassing of anaerobic cultures of *M*. *maripaludis* the following sterile equipment was utilized: 1 mL, 5 mL and 10 mL gas-tight syringes (Injekt®-F, Omnifix®-F, Omnifix®; B. Braun, Melsungen, Germany); hypodermic needles (Gr 14, 0.60 × 30 mm, 23 G × 1 1/4′′; B. Braun, Melsungen, Germany) and cellulose acetate filters with pore size 0.20 μm (LLG Labware, Meckenheim, Germany). A gassing manifold (Taubner and Rittmann, 2016) was used for feeding cultures. A digital manometer (LEO1-Ei, −1…3 bar rel, Keller, Germany) was used for pressure measurements. Serum bottles were shaken or stirred with a magnetic stir bar at 37°C at various rotations per minute (rpm). Assessment of culture growth was carried out by optical density measurements utilizing a spectrophotometer Specord 200 Plus (Analytic Jena, Jena, Germany) at 578 nm (OD_578_).

#### Experimental groups: Shaking vs. stirring

Two distinct methods for agitation of both SB and SCB were compared – shaking and stirring. Shaken closed batch cultures were agitated up to 180 rpm by two devices: an air incubator (ZWYR-2102C; Labwit Scientific, Ashwood, Australia) or an orbital shaker (No. 3019; Gesellschaft für Labortechnik GmbH, Burgwedel, Germany) which was placed in a 37°C climate chamber (TER, CMESS, University of Vienna). Stirred SB and SCB were agitated up to 1,400 rpm by a 25 × 6 mm or a 40 × 8 mm magnetic stir bars, respectively (BRAND GMBH + CO.KG, Wertheim, Germany). Stir bars were added to 141 media prior to sealing and sterilization of anaerobic media containing bottles, which were incubated on stirrer heating plates (2581001; IKA®-Werke GmbH & Co. KG, Staufen, Germany) that were placed either in an air incubator or in a climate chamber (mentioned above) at 37°C.

Both SB and SCB were tested under distinct conditions (shaking or stirring at various rpm) in quadruplicates with a uninoculated control, which was handled identical to the rest of the replicates. Shaken cultures were subjected to agitation rates of 100, 150 and 180 rpm, whereas stirred cultures were agitated at 100, 500, 800, 1,100, 1,400 rpm. Pressure and OD_578_ were measured in regular intervals once a day prior to feeding and further incubation at 37°C. Best experimental groups were reproduced, as a second timepoint for measurements and feed was introduced. For the optimization within each stage (SB or SCB), an inoculum from the stationary phase of the previous experimental group was used. When new series of experiments was started, the first inoculum was acquired from a pre-culture inoculated from cryogenic stocks.

#### Revival of cryogenic stocks

Cryogenic backups were regularly produced from both serum and SCB cultures. For SB, 800 μL of culture and 600 μL of anaerobic 50% (v/v) glycerol in 141 medium were mixed in anaerobic glove box, snap frozen in liquid molecular nitrogen and stored at −70°C. For SCB, 6.67 mL of culture and 5 mL of anaerobic 50% (v/v) glycerol in 141 medium were mixed and handled as mentioned above. Upon revival, backups were slowly thawed on ice, spun down at 2,000 g, supernatant was discarded and pelleted biomass was suspended in 0.8 mL or 6.67 mL freshly prepared 141 medium and inoculated in complemented 141 medium containing SB or SCB, respectively. The first revived generations were not subjected to experiments and were cultivated *via* shaking at 150 rpm as pre-cultures.

### Bioreactors

#### 2.2 L bioreactor setup

*Methanococcus maripaludis* was cultivated with the Eppendorf commercial system DASGIP® Bioblock (76DGTBLOCK) equipped with 4× 2.2 L Bioblock stirrer reactors (Eppendorf AG, Hamburg, Germany) with a working volume of 1.5 L (reactor culture). Temperature was constantly maintained at 37°C. Cooling water was supplied to the off-gas condenser. Sensors for pH and redox potential (59903232 and 105053336, Mettler Toledo, Columbus, OH, United States) were connected to the DASGIP® module and were monitored *via* the company software. Feed solutions of sodium acetate (0.61 mol L^−1^) and Na_2_S·9H_2_O (0.5 mol L^−1^) were prepared in SCB, made anaerobic and maintained anaerobic by connecting them to gas bags filled with a gas mix of H_2_/CO_2_ (4:1). The solutions were supplemented to the reactor vessels through a PTFE tubing (0.8 mm) by the DASGIP® MP4 and MP8 pumps. Gassing flow rate was usually maintained at 0.3 volume gas per volume liquid per minute (vvm) but alternative vvm were also tested for individual experiments (0.15 to 0.6 vvm). CO_2_ was supplemented *via* the DASGIP® MX4/4 mixing module (76DGMX44, Eppendorf AG, Hamburg, Germany) and H_2_ was managed externally *via* the mass flow controller SmartTrak® (C100L; Sierra Instruments, Monterey, CA, United States). The two distinct gas circuits were merged by a three-way junction into a single tubing which directed the gas mix of H_2_/CO_2_ (4:1) through a Millex®-FG filter (SLFG05010; MilliporeSigma, Burlington, MA, United States) into the inflow sparger of the bioreactor vessels. For sampling of cultures, the in-build 370 mm pipe of stainless steel (outer diameter 4 mm, inner diameter 2 mm) was used. Silicon tubing (M0740-2445; Eppendorf AG, Hamburg, Germany) was used for completing and connecting various reactor gas circuits, tubes and ports. Off-gas tubings were directed through a 500 mL SCB (for collection of condensate) and further toward a dedicated opening of a centralized exhaust gas absorption system.

#### 1.5 L reactor cultures: Cultivation and analysis

First, pH and redox probes were calibrated using distinct buffers (pH 7.0/4.1; 10000642/10545151; Fisher Scientific, Hampton, NH, United States) and an ORP solution (240 mV; HI7021L; Hanna Instruments, Woonsocket, RI, United States), respectively. Probes were then plugged into the stirrer reactors filled with 1.434 L of 141 medium without acetate supplementation and the complete reactor setup was sterilized by autoclaving. Then the vessels were positioned into the Bioblock and all circuits were connected and managed through the company software. Temperature was set at 37°C, stirring at 600 rpm and the media was made anaerobic at 0.3 vvm with a flow rate of 20 standard liter per hour (sL h^−1^) H_2_ and 5 sL h^−1^ CO_2_. The medium was supplemented with 30 mL of sodium acetate (0.61 mol L^−1^) *via* injection through a silicone rubber septum and with 6 mL of Na_2_S·9H_2_O (0.5 mol L^−1^) *via* the MP4 pumps. Finally, as a last preparation step before inoculation, the pH was adjusted to 7.00 (± 0.05) with NaOH (10 mol L^−1^) *via* injection through the septum and was not automatically titrated later. Samples for OD_578_ were harvested before and directly after inoculation and at regular intervals during culture growth. After inoculation, feeds of sodium acetate (0.61 mol L^−1^) and Na_2_S·9H_2_O (0.5 mol L^−1^) were supplemented at rates of 0.5 mL h^−1^ and 0.1 mL h^−1^, respectively, and were manually increased upon stable growth. Biomass harvesting was carried out by connecting a needle to the silicon tubing of the sampling pipe and punctuating the butyl rubber of an empty anaerobic SCB with a negative pressure. Biomass from the reactor vessel was directed through the sampling silicon tubing and the needle into the SCB bottle, due to the negative pressure. The collected biomass was then handled in the anaerobic glove box for various purposes: calculations, inoculum preparation, pelleting for storage.

Inoculum preparation for transfer from closed batch to fed-batch cultures was a central subject of this study and various procedures were tested and are discussed later. Here, only the established reproducible methods are described. Biomass (400 mL) from both exponential and stationary phase of SCB or 1.5 L reactor cultures was concentrated by centrifuging at between 2,500 and 4,000 g, mostly at 3,000 g for 15 min at room temperature (RT) using 750 mL bottles and a LH 4000 swing-out rotor (75006475) of a Heraeus 4KR multifuge (75004461, Thermo Fisher Scientific, Waltham, MA, United States). Pelleted biomass from 400 mL starter cultures was then suspended in 30 mL of fresh 141 medium without supplemented acetate and collected into a 50 mL syringe described above and finally injected into reactors.

#### 1.5 L reactor cultures agitation ramps and experimental groups

Bioreactor cultures of *M*. *maripaludis* were subjected to four distinct agitation ramp profiles: “conservative” and “progressive” variations of both stepwise and continuous increase of rpm. The continuous approaches were defined by a constant increase of rpm starting from the inoculation timepoint and differed in their acceleration rate: the conservative and progressive variations reached 600/1,200 rpm after 72 h and 1,200/1,600 rpm after 108 h, respectively. In contrast, the stepwise ramp profiles additionally introduced short steady states when the agitation was maintained at a constant rate. The conservative variation was maintained at 100 rpm 18 h post inoculation and reached 1,200 rpm after 69 h, whereas the progressive was set at 100 only for 6 h and reached the final steady state of 1,500 rpm after 74 h. Additional variations of the above-mentioned ramps have been tested but are not reported here due to lacking positive impact on culture growth.

To organize experiments within this study, a three-digit ID (x.y.z) was assigned to each bioreactor replicate. The digit x defines the phase, which describes bioreactor experiments, starting from an “adaptation” round (first inoculation from SCB into 1.5 L reactor cultures), going through further transfer from stagnating reactor cultures into new reactor cultures until final biomass harvesting. The second digit y describes the consecutive bioreactor run from the beginning of bioreactor experiments within this study and the third digit describes the reactor replicate number on the Eppendorf Bioblock.

#### 22 L bioreactor setup

*Methanococcus maripaludis* was cultivated in a customized 22 L Biostat® C15-3 reactor system (bbi-biotech GmbH, Sautorius Group, Berlin, Germany) with a working volume of 15 L (reactor culture). Rushton turbines were installed at 25, 50 and 80 cm from the bottom of the central agitation shaft. Temperature was constantly maintained at 37°C *via* thermometer and cooling water was supplied to the off-gas condenser. A sensor for pH (104054479, Mettler Toledo, Columbus, OH, United States) was connected to the control unit, and a sensor for redox potential (59904198, Mettler Toledo, Columbus, OH, United States) was operated externally by a multi-parameter transmitter (M300, Mettler Toledo, Columbus, OH, United States). Feed solutions of sodium acetate (0.61 mol L^−1^) and Na_2_S·9H_2_O (0.5 mol L^−1^) were prepared in SCB, made and maintained anaerobic by connecting them to gas bags filled with a gas mix of H_2_/CO_2_ (4:1). The solutions were supplemented to the reactor vessels through a PTFE tubing (0.8 mm) by external pumps (0106141DA0, Watson Marlow Pumps, Falmouth, Cornwall, United Kingdom). Gas flow rate was maintained at 0.3 vvm, as both CO_2_ and H_2_ were managed externally *via* mass flow controllers SmartTrak®. The two distinct gas circuits were merged by a three-way junction into a single tubing which directed the gas mix of H_2_/CO_2_ (4:1) through a filter into the inflow sparger of the bioreactor vessels. For sampling of cultures, a silicone tubing attached to the sampling valve SV-25 was used.

#### 15 L reactor cultures: Cultivation and analysis

First, pH and redox probes were calibrated, as described above. Probes were plugged into their ports and the vessel was filled with 14.490 L of uncomplemented 141 medium and a sterilization cycle was carried out. Additionally, a steam generator Veit 2365/2 (Veit GmbH, Landsberg am Lech, Germany) was used for sterilization of sampling and harvesting valves and of the double mechanical seal. Temperature was set at 37°C, stirring at 600 rpm and the media was made anaerobic at 0.3 vvm with a flow rate of 20 sL h^−1^ H_2_ and 5 sL h^−1^ CO_2_ until pH and redox values remained stable (around 15–20 min). The medium was complemented with 300 mL of sodium acetate (0.61 mol L^−1^) and with 60 mL of Na_2_S·9H_2_O (0.5 mol L^−1^) *via* injection through a silicone rubber septum. Finally, as a last preparation step before inoculation, the pH was adjusted to 7.00 (± 0.05) with NaOH (10 mol L^−1^) *via* injection through the septum and was not automatically titrated later. Biomass (3×400 mL) from stationary phase of 1.5 L reactor culturess was centrifuged at 3,000 g for 15 min at room temperature (RT) using 750 mL bottles and a LH 4000 swing-out rotor (75006475) of a Heraeus 4KR multifuge (75004461, Thermo Fisher Scientific, Waltham, MA, United States). Pelleted biomass was then suspended in 30 mL of fresh uncomplemented 141 medium and collected into 50 mL syringes (3×50 mL) described above and finally injected into reactors. Samples for OD_578_ were harvested before and directly after inoculation and at regular intervals during culture growth. After inoculation, feeds of sodium acetate (0.61 mol L^−1^) and Na_2_S·9H_2_O (0.5 mol L^−1^) were supplemented at rates 5 mL h^−1^ and 1.2 mL h^−1^, respectively, and were manually increased upon stable growth. Biomass harvesting was carried out *via* the harvest and drain valve.

### Best pipeline

The best pipeline combined the most successful experimental groups from each cultivation mode for a continuous scale-up of *M*. *maripaludis* cultivations by transfers of actively growing culture. Unlike the optimization within each cultivation mode where the inoculum was generated from a stagnating culture, for the complete pipeline inoculum was prepared from cultures in their exponential phase. The only exception was the transfer from a 1.5 L reactor culture into the 15 L reactor cultures, where the inoculum was also in stationary phase.

### Calculations

Formula 1 utilizes a linear model (Taubner et al., 2018; Mauerhofer et al., 2021) for calculating the specific growth rate (μ_L_) of *M*. *maripaludis* in closed batch mode, since exponential growth was only documented in 1.5 L reactor cultures, where an exponential formulation (μ) was applied for fed batch experiments (Formula 2). Subsequently, linear and exponential generation times (GT_L_/GT) were calculated from the linear μ_L_ and exponential μ, according to Formulas 3 and 4, respectively. Furthermore, substrate (H_2_/CO_2_) consumption was determined *via* pressure measurements of SB and SCB (Taubner and Rittmann, 2016).


(1) $$\mu_{L}=\frac{\frac{OD_{578}\left(X\right)}{OD_{578}\left(X-1\right)}}{\Delta t}$$

(2) $$\mu =\frac{\ln\frac{OD_{578}\left(X\right)}{OD_{578}\left(X-1\right)}}{\Delta t}$$

(3) $${\mathrm{GT}}_{L}=\frac{2}{\mu_{L}}$$

(4) $$\mathrm{GT}=\frac{\ln\left(2\right)}{\mu }$$

## Results

Within this study *M*. *maripaludis* was cultivated for 4,000 h in 0.05 L serum and 0.4 L SCB cultures, for 3,000 h in 1.5 L reactor cultures and for the first time in a volume of 15 L in a stainless-steel reactor system. Here, an extensive comparison of (linear and exponential) μ, GT and substrate consumption for various agitation and feeding techniques in closed batch mode is presented. Furthermore, reproducible transfer and optimized biomass generation in a fed-batch reactor system is discussed in detail and applied into scale-up pipelines.

### 0.05 L SB cultures

**Figure 1:**
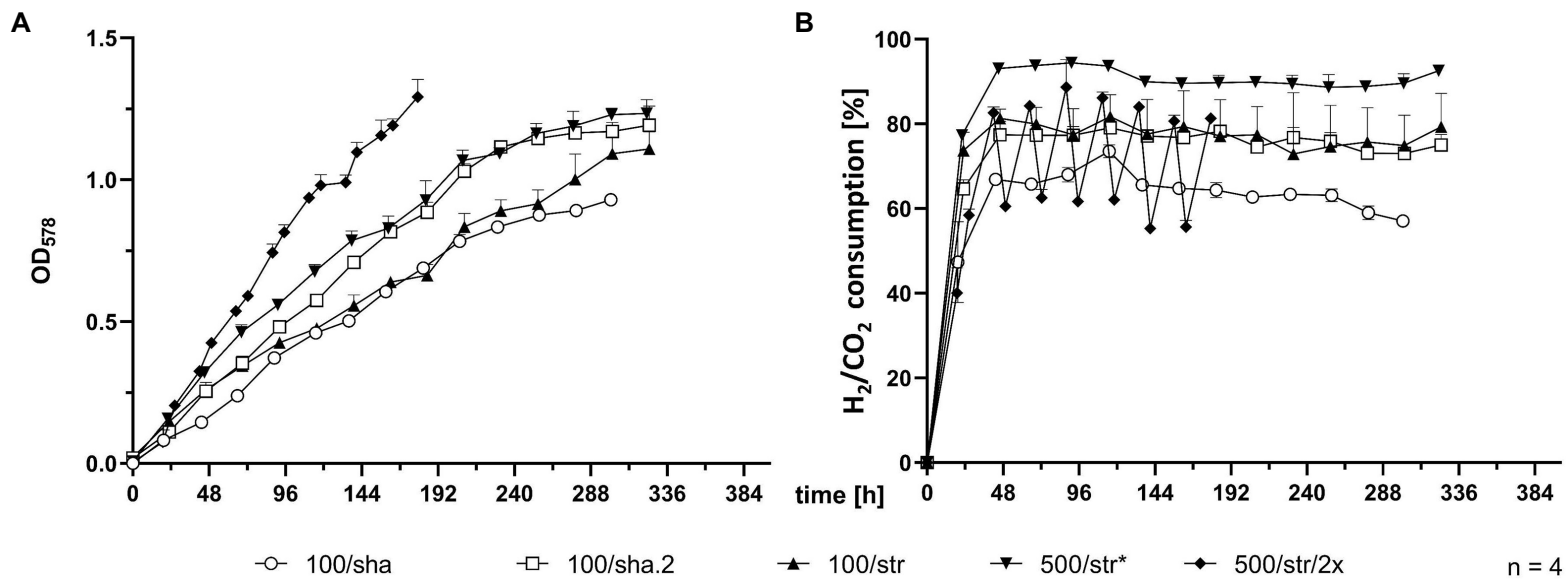
Comparison of 0.05 L SB cultures. **(A)** OD_578_ measured over time in h. **(B)** substrate consumption, measured in % conversion of substrate (H_2_/CO_2_) into product (CH_4_) over time in h. All data points are calculated mean values of biological quadruplicates (*n* = 4). Further details are presented in Table 1. *n = 3 after *t* = 277 h.

SB were subjected to agitation by shaking or by stirring with a magnetic stir bar. Shaking at 100 rpm (100/sha, Figure 1) resulted in the lowest growth (Figure 1A) and substrate consumption (Figure 1B) from all tested groups. Transferring this culture further (100/sha.2) and cultivation under the same conditions showed adaptation and improved GT_L_ (shaker.2, Table 1). Agitating at 100 rpm by stirring (100/str) was more efficient than shaking at the same speed. Stirring at 500 rpm (500/str) displayed a similar growth to adapted 100/sha.2 cultures but reached the highest substrate consumption (Figure 1B) of all tested groups. The best growth in SB was observed upon agitation at 500 rpm by stirring and feeding two times a day (500/str/2×) where the highest maximum OD_578_ of 1.29 (Table 1) was reached around 6 days earlier than all other experimental groups.

**Table 1:** Comparison of culture growth parameters from closed batch experiments.^*^

Closed batch	Agitation		OD_578_		μ_L_ [h^−1^]	GT_L_ [h]
	Method	[rpm]	Max	time [h]		
SB	Shaker	100	0.9292	301	0.07	26.93
	Shaker.2	100	1.1929	325	0.25	7.87
	Stirrer	100	1.1089	325	0.29	6.8
	Stirrer	500	1.2339	323	0.95	2.11
	Stirrer/2×	500	1.2924	179	0.94	2.12
SCB	Shaker	100	1.0819	325	0.21	9.65
	Shaker	180	1.0530	209	0.65	3.06
	Shaker/2×	100	1.0561	291	0.48	4.19
	Stirrer	100	0.4766	326	0.12	16.63
	Stirrer	500	0.9322	321	0.27	7.37
	Stirrer	800	0.9318	158	0.24	8.48
	Stirrer	1,100	0.6804	209	0.15	13.74
	Stirrer	1,400	0.1691	96	–	–

* Closed batch SB und SCB experimental groups are listed with their respective agitation method and intensity (rpm). Maximum (max) measured OD_578_ and the respective needed time are compared. Highest recorded μ_L_ and GT_L_ were calculated according to Formulas 1 and 3. shaker.2, an experimental group, subjected to the same cultivation conditions after inoculation transfer to a new culture; /2×, double H_2_/CO_2_ feeding timepoints.

### 0.4 L SCB cultures

**Figure 2:**
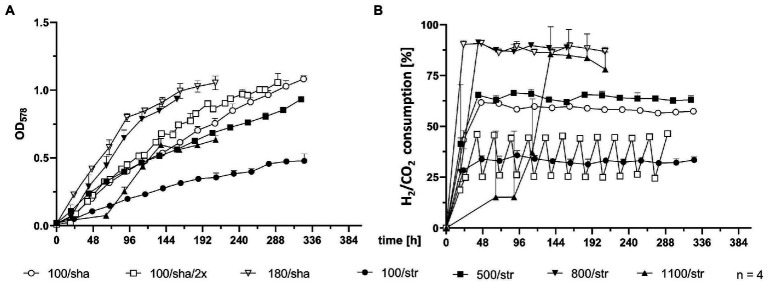
Comparison of 0.4 L SCB cultures. **(A)** OD_578_ measured over time in h. **(B)** substrate consumption, measured in % conversion of substrate (H_2_/CO_2_) into product (CH_4_) over time in h. All data points are calculated mean values of biological quadruplicates (*n* = 4). Further details are presented in Table 1.

SCB were agitated either by stirring with a magnetic stir bar or by shaking. Stirred cultures generally delivered the slowest growth curves, as the experimental groups stirred at 100 (100/str), 500 (500/str) and 1,100 rpm (1,100/str) remained at the bottom of the graph for OD_578_ comparison (Figure 2A). However, cultures which were stirred at 800 rpm (800/str) showed strong improvement of growth and were the second-best experimental group. They also delivered their maximum OD_578_ in the shortest measured time of 158 h (Table 1). Additionally, stirring at maximum possible 1,400 rpm was tested. Quadruplicate cultures reached a mean maximum OD_578_ of 0.169 after 96 h (Table 1). Possible explanations for the inefficient growth at higher agitation intensities are discussed later.

Shaken cultures at 100 (100/sha) and 180 (180/sha) rpm outperformed most of the stirred groups, as the 100/sha group reached the highest OD_578_, and the 180/sha group delivered the lowest GT_L_ of 3.06 h (Table 1). Subjecting 100/sha cultures to double feeding (100/sha/2×) substantially improved growth and reduced the time for reaching maximum culture growth by a day and decreased the GT_L_ more than double (Table 1). The substrate consumption of SCB (Figure 2B) displayed very heterogenous patterns (Figure 2B) and no clear separation between stirred and shaken cultures was observed. Groups 100/str and 100/sha/2× clustered below 50%, 100/sha and 500/str around 60% and 180/sha and 800/str a bit below 90%. Samples from the group 1,100/str showed variating low substrate consumption during the first half of culture growth but reached reproducibly high values during late exponential growth.

### 1.5 L reactor cultures

**Figure 3:**
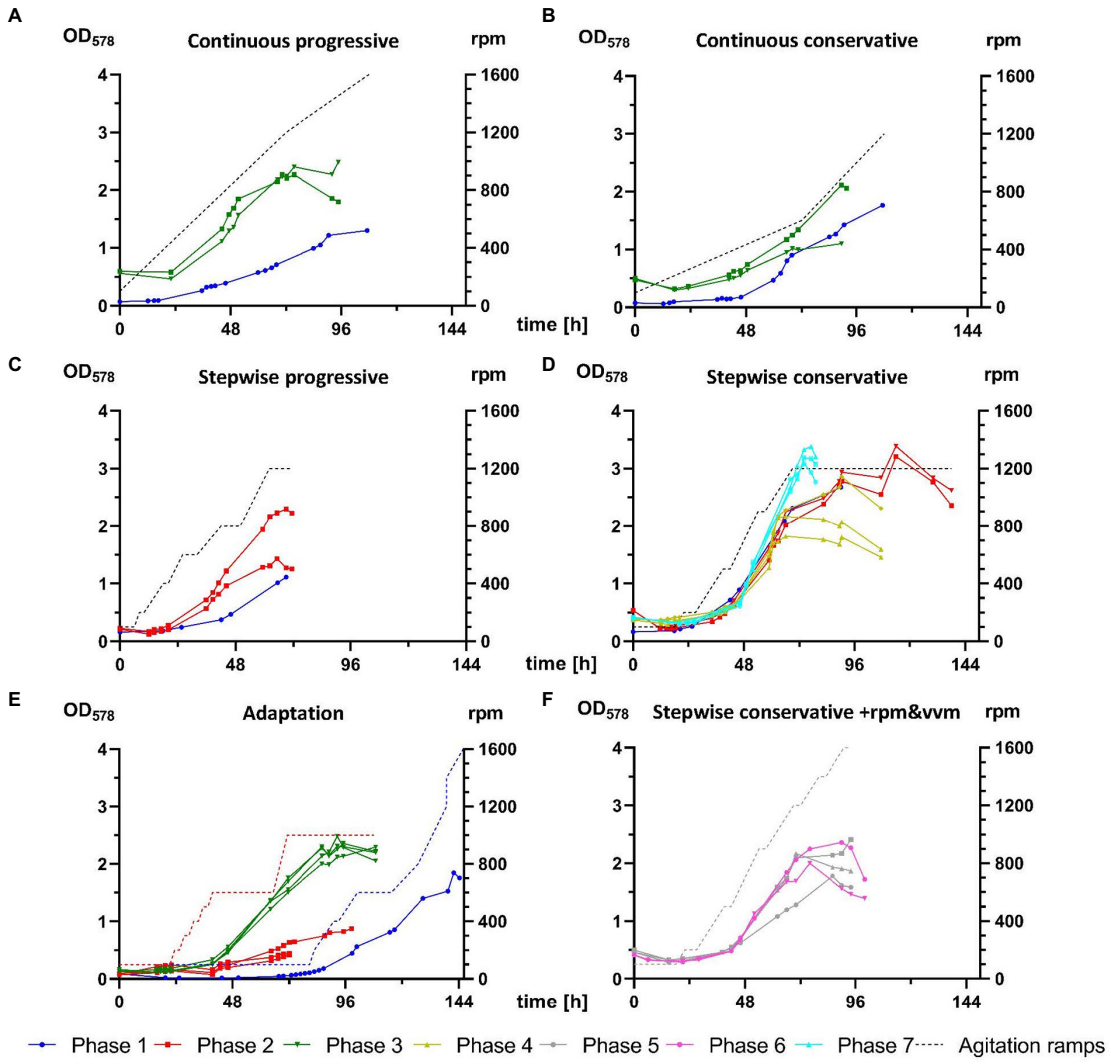
Comparison of *M*. *maripaludis* growth in 1.5 L reactor cultures. OD_578_ (left *Y* axis) measured over time in h for replicates subjected to the following agitation profiles (rpm on right *Y*-axis): **(A)** continuous progressive (Phases 1 and 3), **(B)** continuous conservative (Phases 1 and 3), **(C)** stepwise progressive (Phases 1 and 2), **(D)** stepwise conservative (Phases 1, 2, 4, and 7), **(E)** growth of reactors cultures from Phases 1–3 subjected to adaptation rounds, **(F)** optimization of stepwise conservative ramps by increase of agitation and supply of gaseous substrate (+rpm and vvm) or by increase of agitation and supply of gaseous substrate and decrease of OD_578_ of inoculum (+rpm and vvm-OD) from Phases 5 and 6, respectively. Further details are presented in Table 2.

Phase 1 tested various transfer procedures for inoculation of *M*. *maripaludis* from closed batch cultures into bioreactor vessels. Initial unsuccessful attempts relied on direct injection of 50 mL culture inoculant from SB (at first unwashed, later also spun down and resuspended in fresh 141 medium), which was often grown to low optical densities at low H_2_/CO_2_ pressures, due to the wrong assumption that headspace pressure was an essential adaptation factor. A first successful growth was achieved with the 9^th^ attempt (Figure 3E), where stirring was adjusted manually in response to culture growth. The biomass from this single reactor was continuously transferred in stationary phase of growth and 4 further successful experiments (Figure 3, blue graphs in A–D) were carried out for defining 4 distinct agitation profiles – conservative and progressive variations of continuous and stepwise increase of rpm. At the end of phase 1 several unsuccessful attempts for revival of stored reactor biomass were carried out (data not presented).

Phase 2 adopted a gentler centrifugation at 4,000 g (Table 2) during concentration of biomass upon culture transfer, which resulted in improved survival during the adaptation round, where 3 out of 4 replicates (Figure 3E) exited the lag phase around 48 h earlier than during phase 1. Additionally, those replicates were subjected to a pre-programmed agitation ramp profile (red dotted line, Figure 3E). The biomass was then further transferred in stationary phase for reproducing the stepwise ramp profiles (Figures 3C,D). At this stage the stepwise conservative ramp profile was recognized as the most promising, as all 3 replicates from phases 1 and 2 aligned perfectly and delivered high OD_578_ peaks over 2.5 (Figure 3D).

**Table 2:** Distinct experimental groups of 1.5 L reactor cultures (Figure 3). ^**^

Phase	Runs [N]	n	Inoculum		Main goal	Successful/total	
			OD_578_	g		Runs	Time [h]
1	5	5	Stat.	10,000	Define distinct agitation ramps	6/18	612/1552
2	3	7	Stat.	4,000	Reproduce ramps	3/4	308/399
3	3	8	Exp.*	3,000	Reproduce ramps and test pipeline	3/3	303/303
4	1	3	Late exp. (2.1)		Reproduce best ramp	2/2	107/107
5	1	3	Late exp. (1.8)		Reproduce best ramp + rpm and vvm	2/2	94/94
6	1	2	Exp. (1.3)		Reproduce best ramp + rpm and vvm - OD	2/2	100/100
7	1	3	Exp. (1.5)		Reproduce best ramp -OD	2/2	79/79

**Overview of experimental setup of 1.5 L reactor cultures from Figures 3A–F: Phases; number of runs (N); bioreactor replicates (n); inoculum stage of growth (stat., stationary; exp., exponential), optical density (OD_578_), centrifuge forces (g); main goals of each phase; number of successful/total runs and cultivation time in h. +rpm and vvm: increase of agitation and supply of gaseous substrate; +rpm and vvm-OD: increase of agitation and supply of gaseous substrate and decrease of OD_578_ of inoculum; *exponential only in Figure 3E, not in Figures 3A,B.

During phase 3 for the first time a SCB inoculum in exponential growth (Table 2) was used during the adaptation round. Four reactors were subjected to the agitation ramp from phase 2 (Figure 3E) and grew perfectly aligned almost reaching a true exponential geometry of growth curves and almost matching the best runs from phase 2. This run was in fact the last stage of the proposed best pipeline from this study discussed below. Additionally, *M*. *maripaludis* was transferred as a stationary inoculum to further reproduce the continuous agitation ramps (Figures 3A,B), which in both cases sustained better growth than the single replicates from phase 1.

The main goal of phases 4–7 was to optimize growth of *M*. *maripaludis* upon agitation under the stepwise conservative ramp. Adaptation rounds were carried out unsupervised over the weekend (not shown here) according to the set-up of the adaptation round from phase 3 (Figure 3E). The resulting biomass was used to inoculate single runs (*N* = 1), with either 1 or 2 biological replicates (*n* ≤ 2), which reproduced the stepwise conservative ramp. Phase 4 had an identical setup to the respective experimental groups from phase 1 and 2. During phase 5 the agitation ramp was increased to maximum levels of 1,600 rpm and H_2_/CO_2_ mix was supplied at increasing rates from 0.3 to 2.4 vvm. Phase 6 had the same experimental setup and additionally an inoculum from exponential phase (OD_578_ = 1.3) was used. However, phases 5 and 6 (Figure 3F) were not able to sustain a better growth of *M*. *maripaludis* than during phases 1, 2, and 4.

Finally, the best run in this study was generated during phase 7 (Figure 3D) when the setup of phases 1, 2, and 4 was reproduced in combination with an inoculum from exponential growth, which resulted in the highest recorded OD_578_ value for *M*. *maripaludis* of 3.38 (Table 3). This value was also reached under the same cultivation conditions during phase 1 (Figure 3D) but around 48 h later. Under the stepwise conservative agitation ramps (reactor IDs 2.21.3/2.21.4, 4.27.2/4.27.3/4.27.4) also the highest recorded values for μ and GT of ~0.16 h^−1^ and ~ 4.3 h, respectively, were measured (Table 3).

**Table 3:** Comparison of growth parameters from experiments with 1.5 L reactor cultures.^***^

Replicate ID [Phase.run. replicate]	Ramp	OD_578_		μ [h^−1^]	GT [h]	Wet biomass [g L^−1^]
		Max	Time [h]			
1.9.3	Ad/man	1.8452	142	0.11	6.08	5.17
1.10.2	Cont-cons	1.7652	107	0.12	5.78	7.72
1.10.4	Cont-prog	1.3007		0.10	6.72	4.28
1.12.1	Step-cons	2.6770	90	0.06	11.24	–
1.12.2	Step-prog	1.1111	69	0.06	12.58	–
2.19.2	Ad/step-man	0.4430	72	0.06	11.43	–
2.19.3		0.4180		0.04	16.82	–
2.19.4		0.8757	99	0.10	7.04	–
2.20.1	Step-prog	1.4310	65	0.09	7.02	–
2.20.2		2.2980	69	0.08	8.86	–
2.21.3	Step-cons	3.3884	114	0.06	11.20	3.52
2.21.4		3.2096		0.09	7.86	–
3.23.1	Ad/step-man	2.2960	116	0.10	7.10	–
3.23.2		2.4864	109	0.08	8.78	4.09
3.23.3		2.3598		0.08	8.62	5.96
3.23.4		2.3122		0.10	7.12	6.27
3.24.2	Cont-cons	2.1163	90	0.06	12.58	–
3.24.4		1.1018		0.05	13.82	–
3.25.1	Cont-prog	2.2690	76	0.06	12.36	3.39
3.25.3		2.4040		0.07	9.84	5.39
4.27.2	Step-cons	2.1700	66	0.11	6.34	–
4.27.3		2.8588	91	0.13	5.53	–
4.27.4		1.8230	66	0.16	4.30	–
5.29.1	Step-cons +rpm and vvm	2.1670	70	0.07	10.20	–
5.29.2		2.4113	94	0.06	12.54	–
5.29.4		1.7824	86	0.04	17.78	–
6.31.1	Step-cons +rpm and vvm -OD	2.0025	76	0.09	8.02	–
6.31.2		2.3625	90	0.09	7.30	–
7.33.1	Step-cons. -OD	3.086	74	0.16	4.44	–
7.33.2		3.1852	74	0.14	4.91	–
7.33.3		3.3818	77	0.14	4.89	–

*** Individual reactor replicates are listed with their respective ID ([phase.run.replicate] numbers) and tested agitation ramp (ad: adaptation, man; manual, cont-cons: continuous conservative, cont-prog: continuous progressive, step-cons: stepwise conservative, step-prog: stepwise progressive), +rpm and vvm: increase of agitation and gaseous substrate supply, -OD: decrease of OD of inoculum. For each run a highest recorded (max) OD_578_ and the corresponding time are listed. Specific growth rate (μ) and generation time (GT) were calculated according to Formulas 2 and 4 and compared. If biomass was harvested, the measured weight in grams (g) of wet pellets is presented in the last column on the right. IDs and noteworthy values are matching the color code of respective growth curves from Figure 3.

### Scale-up pipeline

**Figure 4:**
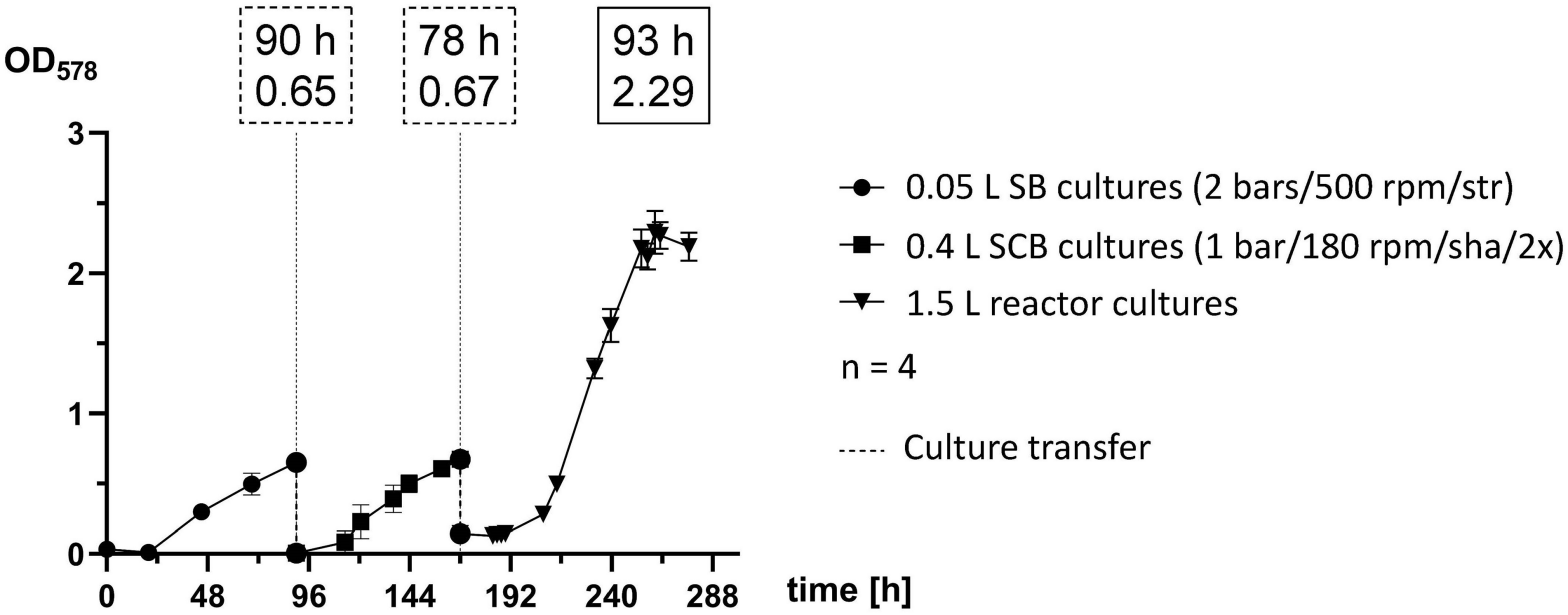
Scale-up pipeline of *M. maripaludis*. OD_578_ (left *Y* axis) measured over time in h for replicates subjected to the following cultivation methods: 0.05 L SB cultures cultivated at 2 bars H_2_/CO_2_ and agitated by stirring at 500 rpm (0–90 h); SCB cultivated at 1 bar H_2_/CO_2_ twice (2×) a day and agitated by shaking at 180 rpm (90–168 h); 1.5 L reactor cultures subjected to adaptation round setup (168–277 h). Dotted line for culture transfer designates harvesting and inoculation procedures between the distinct groups. All data points are calculated mean values of biological quadruplicates (*n* = 4). Dotted text boxes designate time (h) and OD of cultures during transfers. The solid text box designates time (h) of peak OD.

*Methanococcus maripaludis* was inoculated in 0.05 L SB cultures, transferred to 0.4 L SCB cultures and finally into 1.5 L reactor cultures (Figure 4). Each stage of the scale-up pipeline utilized some of the best experimental groups, which were cultivated until reaching exponential phase and then further transferred. SB cultures were agitated by stirring at 500 rpm and were supplemented with gaseous substrate (H_2_/CO_2_) once a day at 2 bars. Quadruplicates reached a mean OD_578_ of 0.6524 after 90 h and were transferred into 0.4 L cultures. The SCB were shaken at 180 rpm and fed twice a day at 1 bar with H_2_/CO_2_. After 69 h (159 h timepoint) they reached OD_578_ of 0.6 but were transferred to 1.5 L cultures after 78 h (168 h timepoint). The growth curve of quadruplicate bioreactor cultures represents the adaptation round of Phase 3 (Figure 3E) and was among the most successful runs at that stage of this study. Exponential growth at OD_578_ of 0.6 was reached after around 48 h (216 h timepoint) and a peak OD_578_ of 2.3 was reached after 92 h (260 h timepoint).

### 15 L reactor culture

**Figure 5:**
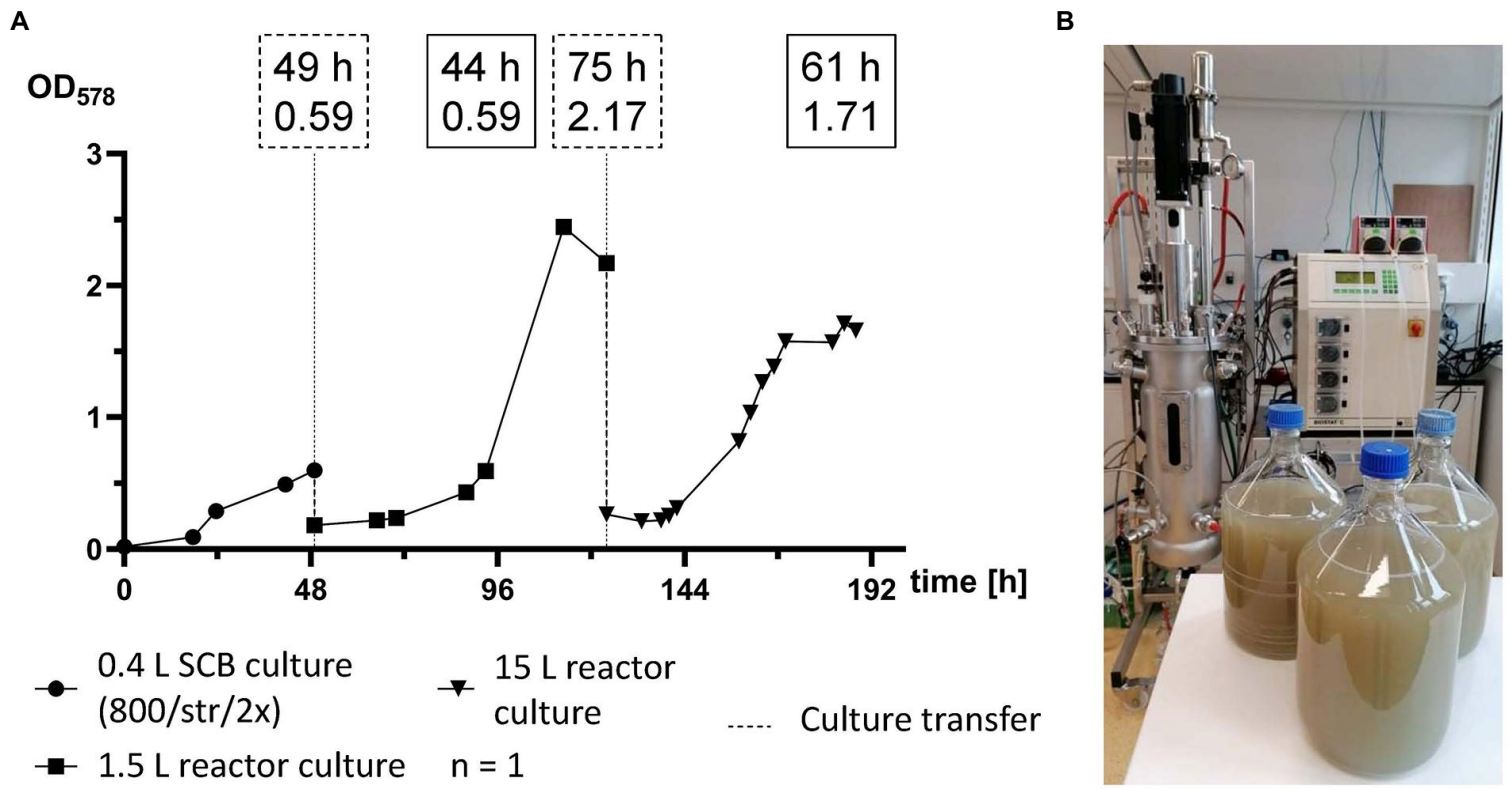
Scale-up of *M*. *maripaludis* toward a 15 L reactor culture. **(A)** OD_578_ (left *Y* axis) measured over time in h for a single replicate subjected to the following cultivation methods: SCB cultivated at 1 bar H_2/_CO_2_ twice (2×) a day and agitated by stirring at 800 rpm (0–49 h); 1.5 L reactor culture subjected to adaptation round setup (49–124 h); a 15 L reactor culture (124–188 h). Dotted line for culture transfer designates harvesting and inoculation procedures between the distinct groups. Dotted text boxes designate time (h) and OD of cultures during transfers. Solid text boxes designate time (h) and OD of crucial stages of culture growth. **(B)** Harvested biomass with the used Sautorius Biostat C 15–3.

For the first time *M*. *maripaludis* was cultivated in a working volume of 15 L in a 22 L stainless steel bioreactor (Figure 5B). For this scale-up experiment, first a 0.4 L pre-culture was prepared in SCB, which was agitated by stirring at 800 rpm and fed twice a day with 1 bar H_2_/CO_2_ (Figure 5). OD_578_ of 0.6 was reached after 49 h (Figure 5A). After biomass harvesting and concentration, a single 1.5 L reactor culture was inoculated and subjected to adaptation round settings. This culture reached exponential growth at OD_578_ of 0.6 after around 44 h (93 h timepoint), peak OD_578_ of 2.4 after 64 h (113 h timepoint) and was further cultivated until stationary phase. After 75 h (124 h timepoint) at an OD_578_ of 2.1, *M*. *maripaludis* was harvested and inoculated into a 15 L reactor culture. There, exponential growth with an OD_578_ of 0.6 was reached after around 25 h (149 h timepoint), maximum growth was recorded at OD_578_ of 1.7 after 61 h (185 h timepoint) and the complete pipeline was completed after 188 h at OD_578_ of 1.66. The 15 L reactor culture produced 5.57 ± 0.39 g L^−1^ wet biomass.

## Discussion and conclusion

Established large scale processes within the microbial biotechnology sector, which are essential for medicine, nutrition and research, are dominated by heterotrophic bacterial and eukaryotic cell factories, which have high carbon footprints, electricity demands and impact on valuable resources (Patel et al., 2020). In contrast, wild type and bioengineered archaea have been strongly neglected and up to date only halophiles have been used for the biosynthesis of commercial products (Pfeifer et al., 2021). However, the demand for high value bioproducts and the urgent need to stop the tremendous exploitation and destruction of natural resources require an immediate biotransformation of the global industry. Harvesting the metabolic potential of genetically tractable autotrophic, hydrogenotrophic, and methanogenic archaea (Lyu and Whitman, 2019) might be a good start.

### Relevance of methanogenic archaea for bioprocess development

Among the archaea, methanogens continue receiving attention due to their biotechnological potential for the production of CH_4_ and other valuable bioproducts (Pfeifer et al., 2021). The documented physiological features of some methanogens, such as high μ (Rittmann et al., 2015; Azim et al., 2017), tolerance to high gas and hydrostatic pressures (ver Eecke et al., 2013; Taubner et al., 2018; Pappenreiter et al., 2019) and resistance to shear forces (Seifert et al., 2014; Azim et al., 2017) clearly make them attractive candidates for cell factories with biotechnological applications. For the development of CH_4_ production bioprocesses from H_2_/CO_2_, volumetric or specific CH_4_ productivity and conversion efficiency have been defined as crucial parameters (Mauerhofer et al., 2018; Rittmann et al., 2018).

However, not all methanogens, that are available as pure cultures, can easily be grown in bioreactors. This and previous studies on the scale-up of methanogens (Mauerhofer et al., 2018) have shown that bioprocess development should focus on examining the type of cultivation method (e.g., closed batch, fed-batch, and continuous culture) and the type of bioreactor (e.g., stirred tank reactor, bubble column, and trickle-bed) that are envisioned for the final production process. Furthermore, identification and optimization of the scaling criteria, the medium, the feed source, quality and quantity, the inoculum storage and preparation, and the type of downstream processing operation are of major importance. For instance, some methanogens grow in inexpensive minimal medium (Taubner and Rittmann, 2016; Mauerhofer et al., 2018) and can be cultivated in standard stirred tank bioreactors (Seifert et al., 2014; Azim et al., 2017; Rittmann et al., 2018).

Methanogens that have been applied for scale-up of CH_4_ or biomass production in bioreactors are *Methanothermobacter marburgensis* and *M*. *maripaludis*, respectively. For *M*. *marburgensis*, successful scale-up to B-TRL ≥5 (Pfeifer et al., 2021) and growth and CH_4_ production in fed-batch, as well as in continuous culture, have been thoroughly examined (Seifert et al., 2014; Azim et al., 2017; Rittmann et al., 2018). However, a pipeline for rapidly producing high biomass concentrations of *M*. *maripaludis* in fed-batch cultivation mode has up to now been missing.

### Expanding opportunities for the cultivation of *Methanococcus maripaludis*

This study provides a comprehensive comparison of various cultivation techniques for *M*. *maripaludis*. By the optimization of established anaerobic methods, an extensive laboratory-scale manual for experimental approach toward rapid biomass generation has been developed. Everyday cultivation of *M*. *maripaludis* has mainly relied on closed batch bottles for decades, as the highest culture volume has increased from 0.2 L to 0.23 L for 36 years (Balch et al., 1979; Goyal et al., 2015). Here, regular growth in 0.4 L SCB cultures supplemented by gaseous H_2_/CO_2_ substrates is being reported, which is equally accessible, reliable and reproducible. Combined with an alternative agitation method by a magnetic stirrer, SCB cultures allow faster and cost-efficient biomass generation which is sufficient for molecular analysis without the need for sophisticated cultivation equipment. This approach was inspired by flexible closed batch systems, developed for formate-utilizing methanogens in culture volumes of 1.5 L (Long et al., 2017). However, here the cultivation of *M*. *maripaludis* in liter ranges relies on gaseous H_2_/CO_2_ substrate supplementation and more sophisticated fed-batch equipment.

In chemostat vessels, *M*. *maripaludis* has been grown in volumes of 1.3 L reaching peak OD_660_ of around 1.7 and the highest reported μ of 0.24 h^−1^ (Haydock et al., 2004), which have almost been reproduced recently (Müller et al., 2021). This system has been established for studies of nutrient limitations (Hendrickson et al., 2007, 2008; Costa et al., 2013), where distinct *M*. *maripaludis* strains (S2, S52) have been cultivated at low optical density in various media (mineral McN, with or without acetate supplementation, but also defined complex McA and McCas) and their OD has been measured at distinct wavelengths (600 or 660), hence complicating straightforward comparison, due to the Lambert–Beer’s law. The application of the chemostat cultivation procedure in a 3 L reactor with 2 L cultures of strain S2, grown in modified rich McCas medium, supplemented with casamino acids, has delivered highest reported OD_600_ of around 2.7 and doubling times of 3 to 4 h (Walters and Chong, 2017). Here, doubling time of 4 to 5 h and the highest OD_578_ of 3.4 have been reached with strain S0001, grown on gaseous H_2_/CO_2_ substrate in reduced 141 medium, supplemented with acetate. Additionally, the first ever reported cultivation of *M*. *maripaludis* in a 22 L stainless steel bioreactor in 15 L working volume introduces novel opportunities for applying wild type and recombinant methanogenic cell factories in biotechnological advances of industry relevant scales.

### 0.05 L SB cultures

At a laboratory scale, closed batch mode of cultivation is usually the everyday method of choice, as it is robust, easy to handle and allows the simultaneous analysis of distinct experimental groups. *M*. *maripaludis* was therefore cultivated only in SB in the authors’ group before the launch of this study. Nevertheless, the generated biomass is rarely sufficient for analytical measurements of physiological processes or for scaling up toward bioprocess development. Here, 0.05 L SB cultures of *M*. *maripaludis* were utilized as a stable platform for testing and comparing different agitation techniques. Instead of two directional shaking in a water bath, SB were either shaken on an orbital shaker or stirred by a magnetic stir in air incubators and in climate chambers. Generally, shaking is the more accessible method, since occupying 4 (or 5 with zero control) magnetic stirrer plates for prolonged period might be problematic. However, considering the geometry of a surface area displacement during highest possible shaking intensities (~180 rpm, here tested only for SCB), this mode of cultivation might have reached the limit of efficiency for transfer of the carbon-containing gaseous phase into the liquid phase within the experiments, carried out in this study.

In contrast, agitating a magnetic stir bar by a digital stirrer plate allows better resolution (1 rpm) and wider range (0–1,400), hence allowing a further increase of vortex surface area to headspace gaseous substrate. Therefore, stirring became the more favorable method for agitation already prior to scaling up to 0.4 L SCB cultures. Interestingly, an adaptation to respective conditions was observed, since subjecting the 100/sha group to the same conditions (100/sha.2) resulted in improved growth, which outperformed 100/str and grouped with the 500/str samples (Figure 1). This might be explained by the positive impact of prolonged exposure to regular feeding, cultivation and sampling intervals. Stirred SB cultures of *M*. *maripaludis* (500/str/2×) delivered highest measured OD_578_ of 1.29 only after being subjected to double feeding timepoints per day (Table 1). However, it remains unclear, whether greater shaking (180 rpm) or stirring (800–900 rpm) would be more favorable for SB cultures of *M*. *maripaludis* in combination with double or more gassing intervals.

### 0.4 L SCB cultures

SCB cultures of *M*. *maripaludis* gradually became the most favorable closed batch technique for cultivation during the progress of this study, since they proved to be as reproducible as SB and additionally, they generated sufficient biomass for culture transfer to fed-batch reactors. Furthermore, reproducible revival of scaled-up cryogenic stocks proved to be efficient for inoculation of pre-cultures, which displayed growth already after 24 h upon cultivation at 150 rpm by shaking (data not shown). Still, SCB cultures have two disadvantages. While SB can be pressurized to up to 5 bar relative pressure (6 bar absolute), SCB can only withstand 1.5 bar relative (2.5 bar absolute) pressure. Therefore, further optimization of SCB cultures must include double or triple feeding timepoints or in the best case rely on automated pressure measurement and feeding system.

The second drawback concerns technical security issues. The weight of a single SCB culture is around 1 kg. Placing 4 (or 5 including a zero control) on a shaker at maximum agitation levels around 180 rpm might damage instruments and even personnel in case of unstable holder installation. Therefore, despite the fact that *M*. *maripaludis* grew best upon agitation at 180 rpm by shaking (Figure 2), during later stages of this study, stirring (at 500 and 800 rpm; Figures 4, 5) was the method of choice, as cultures stirred at 800 rpm performed just slightly worse than shaking at 180 rpm (Figure 2). Identical to SB cultures, increasing the feed to twice a day, improved the growth and reduced GT_L_ two times for SCB cultures shaken at 100 rpm (100/sha and100/sha/2×; Table 1).

Subjecting *M*. *maripaludis* to stirring at 1,100 and 1,400 rpm led to strongly retarded growth. Nevertheless, extreme μ under similar conditions might be possible in combination with pre-adaptation and continuous transfer to increasing agitations. In this context, it was intriguing to observe that the growth curve of SCB cultures, stirred at 1,100 rpm (1,100/str), could only match the growth efficiency of SCB stirred at 500 rpm (500/str) around 113 h post inoculation (Figure 2A) but in terms of substrate consumption (Figure 2B), 1,100/str replicates matched the results of the most successfully growing groups 180/sha and 800/str. Therefore, despite the need for preadaptation and the apparent lower rate of biomass generation of unadapted cultures, stirring at higher rpm obviously allows better transfer of gaseous substrate into the media and higher rate of methanogenesis by less dense cultures.

### 1.5 L reactor cultures

The optimization of closed batch conditions was mainly motivated by the need to generate sufficient biomass for inoculation into bioreactor vessels, since initial bioreactor inoculations unsuccessfully attempted to utilize 0.05 L SB culture biomass (not shown in results). Stirred 0.4 L SCB cultures were later established as the most promising pre-culture due to magnetic stir bars mimicking the mechanical pressure of the Rushton impellers in bioreactor vessels. However, inoculum from SCB cultures shaken at 150 rpm was also regularly grown successfully in bioreactor cultures during unsupervised adaptations rounds from phases 4–7 (data not shown).

During crucial stages of this project, softer harvesting procedure proved to be the most important aspect for reproducible growth in a fed-batch mode. In phase 1, inoculum harvesting was carried out at 10,000 g (Table 2), leading to prolonged lag phase and slow linear growth in all tested ramps (Figures 3A–E). Reducing the g force to 4,000 (phase 2) and further to 3,000 (from phase 3 on) and reproducing the experiments from phase 1, substantially improved culture growth, which is visible by the higher starting OD_578_, due to enhanced cell survival, and by improved growth curves and higher peak maxima (Figures 3A,B,D).

The growth of *M*. *maripaludis* in 1.5 L reactor cultures was further improved as an inoculum in exponential phase was used for the first time in the adaptation round of phase 3 (Figure 3E). The same impact was observed in the case of the most successful stepwise conservative ramp profile (Figure 3D), which was reproduced several times (phases 1, 2, 4, and 7) where the best growth was recorded during phase 7 when for the first time an exponential inoculum was used. However, subjecting biomass in late and early exponential phase of growth to alternative stepwise conservative ramp profiles during phases 5 and 6, respectively (Figure 3F), did not compensate the increase of rpm above 1,200 rpm. The increase of rpm to highest possible levels aims at better solubility of gaseous H_2_/CO_2_ substrate into the liquid phase but obviously there is an upper limit for *M*. *maripaludis*, which is supported by reports of its rather fragile cellular structure. In combination with SCB data, results of this study suggest a value around 900 rpm (±100). Nevertheless, the use of inoculum in exponential growth has the potential to improve culture performance of *M*. *maripaludis* upon exposure to continuous and stepwise progressive ramp profiles, as those conditions were only tested with an inoculum in stationary phase. The reason for that is, that all 1.5 L reactor culture experiments of this project were carried out on a single reactor unit with 4 replicate vessels, meaning that inoculum could be generated only after completion of the previous cultivation round.

Despite the fact that cultivation time for closed batch has been massively reduced and fed-batch cultures of *M*. *maripaludis* have surpassed highest reported OD_578_ values, much can be further improved. Regarding a best agitation ramp profile for 1.5 L reactor cultures, it seems that a continuous progressive increase supports the quickest exit of the lag phase after 24 h and sustains exponential growth until 48 h, where the increase of rpm above 800 becomes too stressful for *M*. *maripaludis* and marks the beginning of the stationary phase (Figure 3A). Therefore, subjecting exponential inoculum to a continuous progressive ramp ending at 800–1,000 rpm might be more favorable than a stepwise conservative ramping. A second-generation batch culture, which has been adapted to higher agitations, might even withstand a transfer into a culture with a constant agitation at 600–800 or alternatively into a culture with starting agitation at 400–600 (for 24 h), followed by continuous increase (over 12–24 h) to 800–1,000 rpm.

### Scale-up pipelines

The proposed scale-up pipeline of *M*. *maripaludis* does not necessarily include the best performing experimental groups for each stage but rather a flexible combination of cultivation methods. As discussed earlier, agitation at great intensities (≥500 rpm) in closed batch mode might require pre-adaptation for peak performance of *M*. *maripaludis*, which would be the case for applied projects, aiming at high productivity of an archaeal cell factory. On the other hand, biomass generation for fundamental studies can easily adopt the experimental setup, presented here (Figure 4). Depending on the availability of equipment, fellow researchers may recombine cultivation techniques in closed batch mode and agitation ramps upon prolonged continuous transfer in fed-batch mode.

Furthermore, the first ever reported 15 L reactor culture of *M*. *maripaludis* would support scaling up of biomethanation processes toward industry relevant dimensions. Next steps would require using an inoculum in exponential growth and comparison of distinct agitation ramp profiles.

In summary, this study provides a comprehensive manual of diverse techniques for the cultivation of the autotrophic, hydrogenotrophic methanogen *M*. *maripaludis*. The optimization of established methods and the development of novel strategies can be also adapted for other autotrophic organisms. In terms of closed batch mode, increasing the number of feeding timepoints and testing agitation at moderate high intensities (800–1,000 rpm) would easily define conditions for peak performance. Single stage fed-batch cultivation of *M*. *maripaludis* would require additional experiments for defining best cultivation conditions and can be further advanced toward a continuous culture. Developing reproducible growth in a 15 L reactor culture would generally follow the experimental approach for 1.5 L reactor cultures by testing most favorable agitation ramp profiles and applying inoculum in exponential growth.

## Data availability statement

The raw data supporting the conclusions of this article will be made available by the authors, without undue reservation.

## Author contributions

HP and SK-MRR conceived and designed the study, and wrote the manuscript. HP, AR, LP, and DN performed the research. HP, AR, and LP analyzed the data. HP, BR, and SK-MRR contributed new methods or models. All authors contributed to the article and approved the submitted version.

## Conflict of interest

AR, BR, and SK-MRR declare to have competing commercial and financial interests due to their employment in the Arkeon GmbH.

The remaining authors declare that the research was conducted in the absence of any commercial or financial relationships that could be construed as a potential conflict of interest.

## Publisher’s note

All claims expressed in this article are solely those of the authors and do not necessarily represent those of their affiliated organizations, or those of the publisher, the editors and the reviewers. Any product that may be evaluated in this article, or claim that may be made by its manufacturer, is not guaranteed or endorsed by the publisher.
